# Coexistence of normal and inverse deuterium isotope effects in a phase-transition sequence of organic ferroelectrics†

**DOI:** 10.1039/c9ra06489c

**Published:** 2019-12-02

**Authors:** Sachio Horiuchi, Shoji Ishibashi, Kensuke Kobayashi, Reiji Kumai

**Affiliations:** Electronics and Photonics Research Institute (ESPRIT), National Institute of Advanced Industrial Science and Technology (AIST) Tsukuba Ibaraki 305-8565 Japan; Research Center for Computational Design of Advanced Functional Materials (CD-FMat), National Institute of Advanced Industrial Science and Technology (AIST) Tsukuba Ibaraki 305-8568 Japan; Condensed Matter Research Center (CMRC) and Photon Factory, Institute of Materials Structure Science, High Energy Accelerator Research Organization (KEK) Tsukuba Ibaraki 305-0801 Japan

## Abstract

Supramolecular cocrystals of anilic acids with 2,2′-bipyridines exhibit successive phase transitions as well as unusual isotope effects. Ferroelectricity driven by a cooperative proton transfer along the supramolecular chains is accompanied by huge permittivity (a maximum of 13 000) at the Curie point, as well as a large spontaneous polarization (maximum 5 μC cm^−2^) and a low coercive field ranging from 0.5 to 10 kV cm^−1^. Deuterium substitutions over the hydrogen bonds smoothly raise the Curie point and simultaneously reduce other phase-transition temperatures by a few tens of degrees. The coexistence of opposite isotope effects reduces the temperature interval of the intermediate paraelectric phase from 84 to 10 K for the 5,5′-dimethyl-2,2′-bipyridinium bromanilate salt. The bipyridine molecules exhibit interplanar twisting, which represents the order parameter relevant to the high-temperature phase transitions. The normal and inverse temperature shifts are ascribed to the direct and indirect effects, respectively, of the lengthened hydrogen bonds, which adjusts the molecular conformation of the flexible bipyridine unit so as to minimally modify their adjacent intermolecular interactions.

## Abstract 2

Deuterium substitutions of the hydrogen-bonded ferroelectrics smoothly raise the Curie point and simultaneously reduce other phase-transition temperatures by a few tens of degrees.

## Graphical abstract

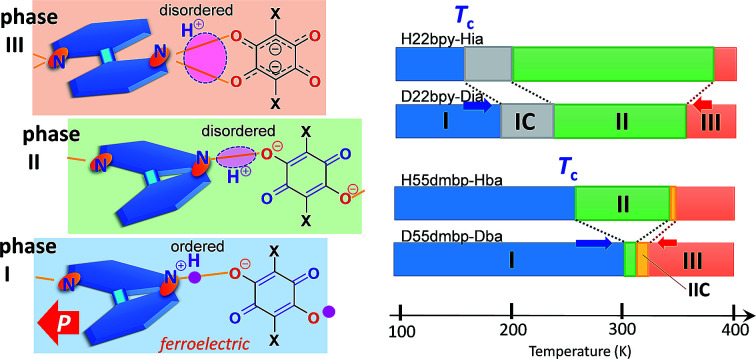

## Introduction

The mobility of protons over hydrogen bonds often triggers various types of structural transformation in the solid state. The most fascinating examples include ferroelectrics and antiferroelectrics, in which crystal structures and the associated electric polarization are transformed in response to such external stimuli as changes in temperature, pressure, stress, or electric fields.^1,2^ These strong couplings among electric, mechanical, and thermal inputs and outputs have been utilized in capacitors, sensors, memories, actuators, and optoelectronics devices, among others.^3,4^

Other intriguing issues pertaining to hydrogen-bonded compounds are related to the replacement of hydrogen by deuterium, because the distinct quantum–mechanical properties of deuterium often change the potential-energy curves of hydrogen bonds and alter proton dynamics sufficiently to have marked effects on the relative stability of phases and on electric, elastic, and optical properties, as well as on hydrogen-bond geometries.^5–11^ These effects have been observed in many inorganic and organic compounds, including the KH_2_PO_4_ (KDP) family, squaric acid, and 9-hydroxyphenalen-1-one, in which proton dynamics play crucial roles in polarization- and phase-switching, as evidenced by the huge isotope effects observed upon deuteration of the hydrogen bonds.^12–17^

**Chart 1:**
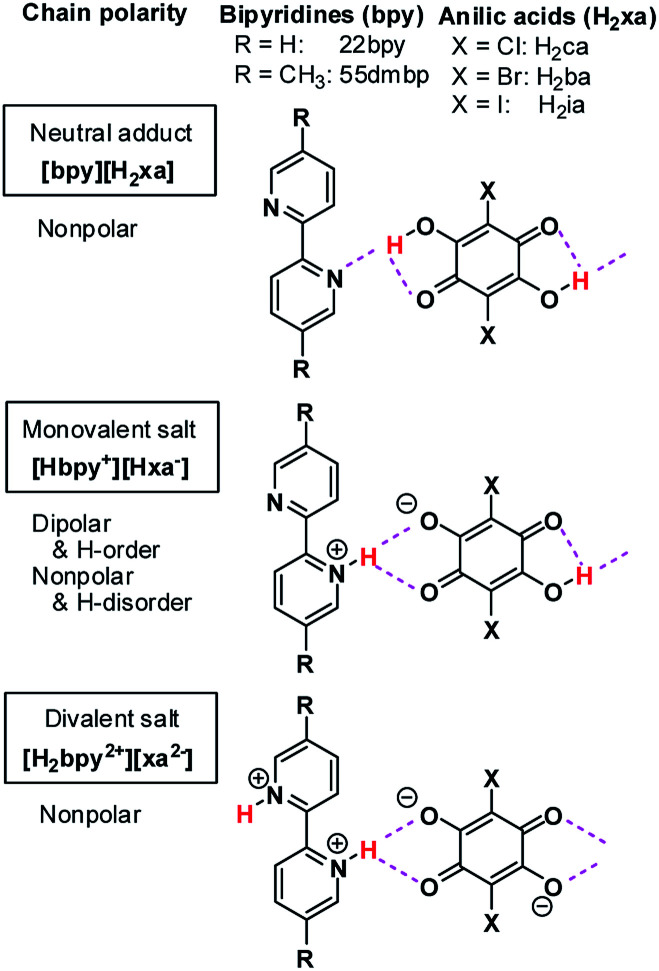
Haloanilic acids, 2,2′-bipyridines, and their supramolecular chains of different valence states.

Over the last decade, similar microscopic principles have been increasingly applied in the design of high-performance ferroelectric organic solids for use in environmentally benign electronic devices.^18–26^ One successful approach has been to accommodate mobile protons between proton-donating (D) and proton-accepting (A) moieties or molecules, as exemplified by the discovery of ferroelectric cocrystals of phenazine (Phz) with the haloanilic acids (2,5-dihalo-3,6-dihydroxy-*p*-benzoquinones) chloranilic acid (H_2_ca) or bromanilic acid (H_2_ba).^27^ These haloanilic acids, designated as H_2_xa, are strong dibasic acids suitable for realizing ferroelectricity. As shown in Chart 1, two-step deprotonation of the hydroxy groups transforms the π-conjugated molecular symmetry as well as the charge state from neutral H_2_xa through the Hxa^−^ monoanion to the xa^2−^ dianion. Whereas H_2_xa and xa^2−^ are nonpolar, Hxa^−^ monoanions display bistable polarities, as they can be polarized in either of two opposing directions, depending on the location of the proton. Their counterparts are nitrogen-containing aromatic compounds such as Phz, various 2,2′-bipyridine derivatives, 2,3-di-2-pyridinylpyrazine (dppz), or 1,5-naphthyridine (npd), which have moderate basicities. The presence of two more nitrogen atoms in each molecule of the base permits the acid and base molecules to self-assemble alternately to form linear supramolecular chains.^28–30^ The resulting series of acid–base cocrystals provide a suitable platform for achieving a deeper understanding of how hydrogen-bonded structures show changes in their microscopic ferroelectric properties and related macroscopic properties through the ordering or dynamics of protons. Earlier studies on some ionic crystals found some analogous behaviors with the KDP family; deuterium substitution elongated their hydrogen-bond length, and increased the potential-energy barriers for proton dynamics.^28,31–33^ The relative stabilization of the proton-ordered ferro/antiferroelectric state raises the Curie point as well as the ferroelectric polarizations.

Here we report some exotic isotope effects and successive phase transitions in new supramolecular ferroelectrics consisting of anilic acids and 2,2′-bipyridine derivatives (Chart 1). Deuterium substitution over the hydrogen bonds smoothly raises the Curie point and simultaneously lowers other phase-transition temperatures by a few tens of degrees. The coexistence of opposing isotope effects reduces the temperature separation of the two phase transitions from 84 K to 10 K. The bipyridines exhibit molecular flexibility through interplanar twisting, which turns out to be relevant to the high-temperature phase transitions. The inverse temperature shift originates from an indirect effect of the hydrogen-bond geometry, which adjusts the molecular conformation of the bipyridine unit upon deuteration so as to minimize the changes in adjacent intermolecular interactions.

## Results and discussion

### General properties of materials

First, let us review the basic properties of the ferroelectric cocrystals as an introduction to the physical and structural assessments reported below.

#### Materials

The 1 : 1 cocrystal of 2,2′-bipyridine (22bpy) and iodanilic acid (H_2_ia) is a new compound that crystallizes as a proton-transferred monovalent salt. As reference compounds for our study we chose two compounds with similar crystal structures; these were cocrystals of 5,5′-dimethyl-2,2′-bipyridine (H55dmbp) with Hia^28^ and with Hba,^34^ the ferroelectricity of which has been reported independently. All these single crystals can be satisfactorily grown through slow evaporation of solutions in methanol, and they comprise linear supramolecular chains of alternating acid and base molecules, as demonstrated below. The use of CH_3_OD (99.5% D) solutions gave the corresponding deuterium-bonded D55dmbp-Dba (degree of deuteration: 94%), D55dmbp-Dia (93%), and D22bpy-Dia (85%) crystals (Fig. S1†). We also studied the thermal properties of the iodanilate salt of fully deuterated 2,2′-bipyridine (22bpy-*d*_8_), prepared as a reference material.

#### Emergence of multiple phases

The ferroelectric crystals examined exhibit many common structural phases and characteristics. The low-temperature Phase I is the ferroelectric ground state. Phase II is a paraelectric state that appears over wide temperature range, including room temperature. In the high-temperature region, the crystals, except for H55dmbp-Hia, display an additional paraelectric state, which we will refer to as Phase III. There are some differences in the detailed phase-transition sequences, in that the H22bpy-Hia and H55dmbp-Hba crystals display additional intermediate phases denoted IC and IIC. For both compounds, deuterium substitution dramatically modifies the phase-transition temperatures, although the isomorphous phase-transition sequences and electric properties are retained, as discussed below. The main structural phases, Phase I, Phase II, and Phase III, were characterized by means of temperature-variable X-ray diffraction studies on D22bpy-Dia and H55dmbp-Hba single crystals as models for their isotropic counterparts.

#### Ground-state crystal structures

**Fig. 1:**
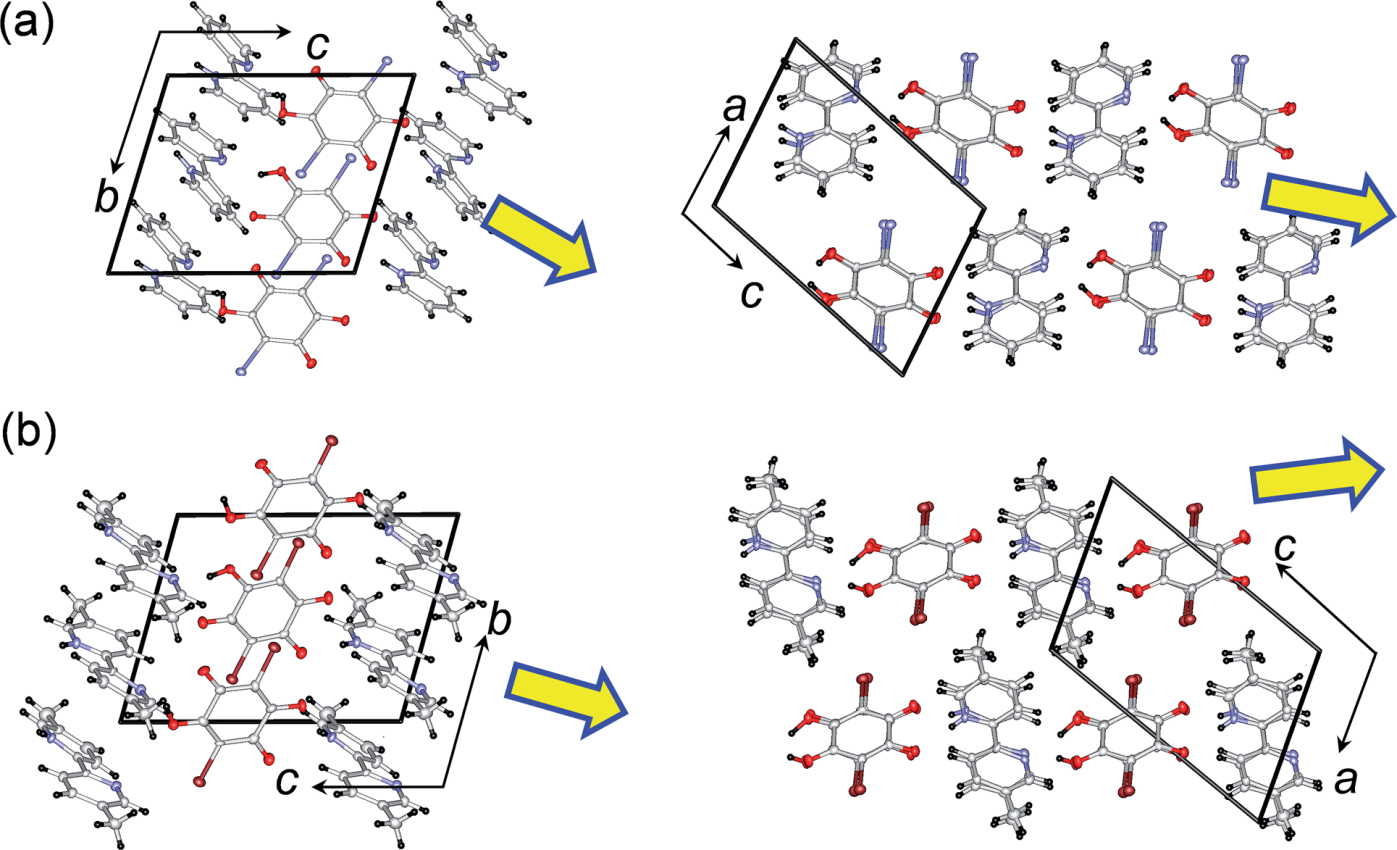
Crystal structures of supramolecular ferroelectrics in the low-temperature polar Phase I. (a) D22bpy-Dia at *T* = 100 K. (b) H55dmbp-Hba at *T* = 173 K. The molecular arrangements projected along the crystallographic *a* and *b* directions are depicted in the top and bottom panels, respectively. Thick open arrows point to the theoretical direction of spontaneous polarization.

Before we discuss the anisotropic electric properties, this section outlines the molecular packings in the ferroelectric Phase I for the H22bpy-Hia and H55dmbp-Hba crystals. Each crystal lattice consists of two formula units and belongs to the triclinic polar space group *P*1. As shown in Fig. 1, the protons are unidirectionally ordered in alternating N⋯H–O and N–H^+^⋯O^−^ hydrogen bonds, which is typical for a monovalent salt at low temperatures.

These two crystal structures appear to have very similar molecular arrangements when viewed along the crystal *a* or *b* axis, as well as similar unit-cell parameters. Whereas the H55dmbp-Hba is isomorphous with ferroelectric H55dmbp-Hia, H22bpy-Hia shows a trivial difference in the orientation of the base molecules in the *b*-axis projection (Fig. 1b). The intermolecular hydrogen-bonds form a straight chain of alternating acid and base molecules, each of which form a separate π–π stack. In the H22bpy-Hia and H55dmbp-Hxa crystals, the chains are oriented parallel to the [212] direction. Note that the molecules are stacked with a doubled periodicity along the *b*-direction.

### Dielectric properties

The dielectric permittivity and polarization hysteresis were examined for the H22bpy-Hia and H55dmbp-Hba crystals as well as for their deuterated analogues. Below, we describe the anisotropic and thermal behaviors of the former two crystals, as well as the effects of deuteration, before discussing theoretical simulations of electric polarizations in comparison with the experimental ones.

#### Dielectric anisotropy

**Fig. 2:**
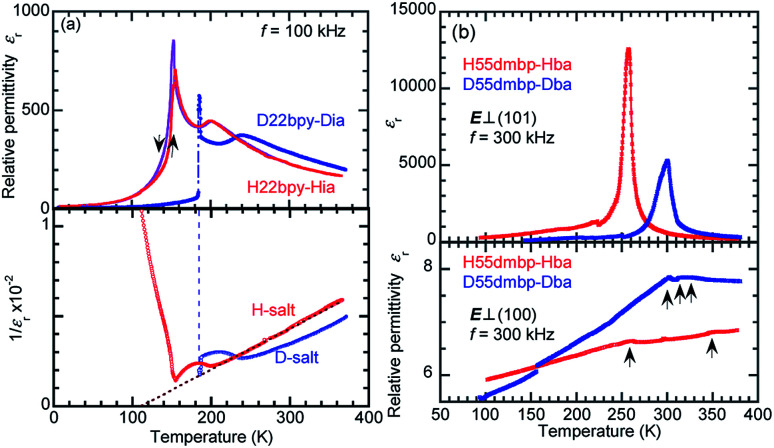
Temperature dependence of dielectric properties. (a) The relative permittivity *ε*_r_ (top) and its inverse *ε*_r_^−1^ (bottom) measured with an ac field (100 kHz) applied in the direction normal to the crystal (101) planes of H22bpy-Hia and D22bpy-Dia single crystals. (b) The relative permittivity *ε*_r_ of H55dmbp-Hba and D55dmbp-Dba single crystals. The directions of the applied ac electric field *E* (300 kHz) are approximately parallel [*E*_⊥_ (101) plane; top panel] and normal to that of hydrogen-bonded chain (bottom).

**Fig. 3:**
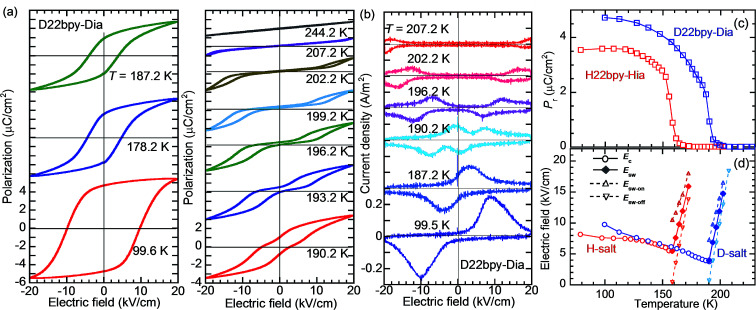
Temperature variation of the polarization-switching properties of the H22bpy-Hia and D22bpy-Dia crystals. (a) Electric polarization (*P*) *versus* electric field (*E*) hysteresis loops of a D22bpy-Dia single crystal at temperatures below *T*_c_ (left) and above *T*_c_ (right). (b) Corresponding current density (*J*) *versus E* curves. A triangular waveform voltage of frequency 1 Hz was applied normal to the crystal (101) plane. (c) The remanent polarization. (d) The coercive field (*E*_c_) in the ferroelectric phase and the forward (*E*_sw-on_), backward (*E*_sw-off_), and average (*E*_sw_) switching fields in the paraelectric phase. These fields were obtained at the current peaks in the *J*–*E* curves.

**Fig. 4:**
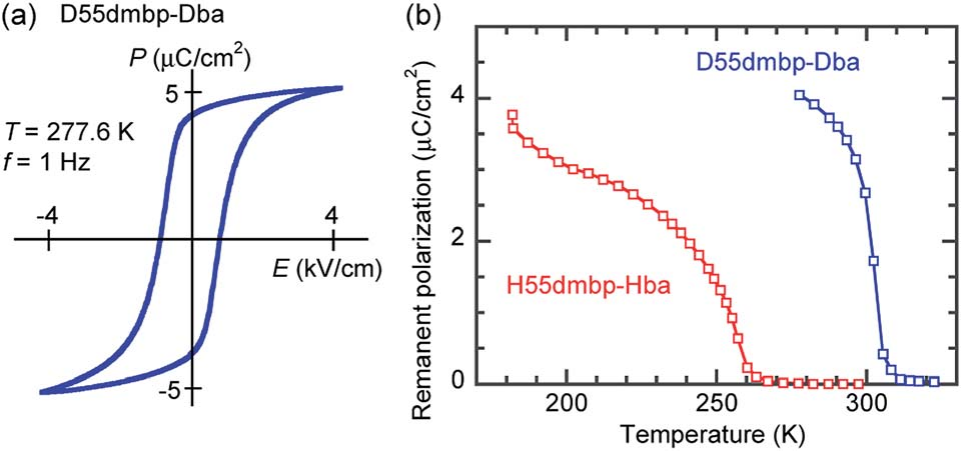
Ferroelectric properties of H55dmbp-Hba and D55dmbp-Dba single crystals measured with a triangular waveform voltage of frequency 1 Hz applied normal to the crystal (101) plane. (a) *P*–*E* hysteresis loop with the best remanent polarization on the deuterated salt. (b) Temperature dependence of the remanent polarizations.

The dielectric response of H22bpy-Hia and H55dmbp-Hba crystals is highly anisotropic (Fig. 2; S2 and S3†) because of the low symmetry of the molecular and crystal (triclinic) structures. The permittivity is maximal when the direction of the applied ac electric field is approximately parallel to the hydrogen-bonded chains. In contrast to the huge response along the supramolecular chains as described below, the relative permittivity is less than 8 in both lateral directions over the entire temperature range. The dielectric anisotropy is also temperature-dependent and emphasized at the ferroelectric transition point. Note that the presence of a few kink-like anomalies suggests successive phase transitions (see Fig. S2 and S3†). The strong anisotropy is manifested as a confinement of the electric polarity in one dimension parallel to the supramolecular chains. Therefore, the argument below focuses on the dielectric response along the chains. The temperature-dependent relative permittivity (Fig. 2) and polarization hysteresis (Fig. 3 and 4) reveal the dipolar-fluctuation and spontaneous-polarization characteristics, respectively, of the ferroelectric phase transition. The triclinic symmetry inevitably causes a slight deviation in the field direction *E*_⊥_ (101) plane from the [212] direction of the chain. Whereas the H22bpy-Hia crystal has a tilting angle of 18.1° (Fig. 2), the H55dmbp-Hba and H55dmbp-Hia crystals exhibit corresponding deviations of 23.8° and 14.5°, respectively.

#### H22bpy-Hia

The relative permittivity *ε*_r_ of H22bpy-Hia is as large as 200 at room temperature (Fig. 2a). A cusp-like peak with a maximum *ε*_r_ of about 800 at low temperatures corresponds to the Curie point *T*_c_. The thermal hysteresis and discontinuous jump in permittivity at *T*_c_ are manifestations of the first-order phase transition. Over a wide temperature range that includes room temperature, the permittivity is characteristic of a paraelectric material, obeying the Curie–Weiss law *ε*_r_^−1^ = (*T* − *θ*)/*C*, where *C* and *θ* are the Curie–Weiss constant and the Weiss temperature, respectively. These parameters (Table 1) are obtained from a least-squares fit of the *ε*_r_^−1^ – *T* data (Fig. 2a). A clear kink-like peak at *T*_A_ = 201 K suggests the emergence of an additional phase between paraelectric Phase II and ferroelectric Phase I; hereafter, this intermediate phase is referred as Phase IC.

**Table 1:** Dielectric properties of ferroelectric supramolecules of anilic acids

Compound^a^	Space Gr. of FE (PE) structure	*T*_c_ (K) {Δ*T*_c_ (K)}	Permittivity		Ferroelectric polarization (μC cm^−2^)		Ref.
			*C* (K)	*θ* (K)	Experimental *P*_r_ (or *P*_s_)	Theoretical^b^ |*P*| (*P*_*x*_, *P*_*y*_, *P*_*z*_)	
**Ionic supramolecules**							
1h H22bpy-Hia	*P*1 (*P*1̄)	158	4.49 × 10^4^	111.1	3.6⊥(101)	—	—c
1d D22bpy-Dia	*P*1 (*P*1̄)	190 {+32}	5.88 × 10^4^	92.9	4.7⊥(101)	8.21 (1.77, 1.72, 7.83)	—c
2h H55dmbp-Hia	*P*1 (*P*1̄)	268	1.39 × 10^4^	251	4.2⊥(101)	6.56 (0.76, 2.77, 5.90)	20 and 28
2d D55dmbp-Dia	*P*1 (*P*1̄)	335 {+67}	2.26 × 10^4^	319	4.0⊥(101)	—	20 and 28
3h H55dmbp-Hba	*P*1 (*P*1̄)	259	2.43 × 10^4^	258.4	3.6⊥(101)	7.22 (3.73, 0.37, 6.17)	—c
3d D55dmbp-Dba	*P*1 (*P*1̄)	303 {+44}	1.9 × 10^4^	299	4.2⊥(101)	—	—c
4 H66dmbp-Hca	*P*2_1_ (*P*2_1_/*c*)	378^d^	—	—	8∥[010]	9.94 (0, 9.94, 0)	30
5 Hdppz-Hca	*Cc* (*C*2/*c*)	402	—	—	5.2∥[001]	7.51 (0.08, 0, −7.51)	29
6 Hdppz-Hba	*Cc*	>420	—	—	5.8∥[001]	—	29
7h H_2_tppz-(Hca)_2_	*Cc* (*C*2/*c*)	172	—	—	0.062∥[110]^e^	—	32
7d D_2_tppz-(Dca)_2_	*Cc* (*C*2/*c*)	240 {+68}	—	—	—	—	32
8h H_2_tppz-(Hba)_2_	*Cc* (*C*2/*c*)	334	39.5	333.3	0.12∥[110]^e^	—	32
8d D_2_tppz-(Dba)_2_	*Cc* (*C*2/*c*)	365 {+31}	51.9	363.4	∥[110]	—	32

**Neutral supramolecules**							
9h Phz-H_2_ca	*P*2_1_ (*P*2_1_/*n*)	253	4.96 × 10^3^	253	1.7∥[010]^e^	(0, 0.55, 0)	27
9d Phz-D_2_ca	*P*2_1_ (*P*2_1_/*n*)	303.5 {+50}	5.02 × 10^3^	301.2	1.7∥[010]^e^	—	31
10h Phz-H_2_ba	*P*2_1_ (*P*2_1_/*n*)	138	3.97 × 10^3^	137.4	0.9∥[010]^e^	—	27
10d Phz-D_2_ba	*P*2_1_ (*P*2_1_/*n*)	204 {+66}	4.60 × 10^3^	201.6	1.2∥[010]^e^	—	31
11 Phz-H_2_fa	*P*2_1_ (*P*2_1_/*n*)	>0.6 GPa^f^	4.68 × 10^3^	190.7	—	—	33
12 dppz-H_2_fa	(*C*2/*c*)	>0.3 GPa^f^	—	—	—	—	29

a dppz = 2,3-di-2-pyridinylpyrazine; tppz = 2,3,5,6-tetra-2′-pyridinylpyrazine; 66dmbp = 6,6′-dimethyl-2,2′-bipyridine; H_2_fa = fluoranilic acid; see Chart 1 for abbreviations of other chemicals.

b The Cartesian coordinate system (*x*, *y*, *z*) was chosen to be parallel to (*a*, *b*′, c*) and (*a*, *b*, c*) for the triclinic and monoclinic crystallographic axes, respectively, where the *b*′-direction is taken as perpendicular to the *a* and c* axes.

c This work.

d Depolarization temperature.

e Spontaneous polarization values obtained from measuring the pyroelectric current.

f The ferroelectric phase appears only at high pressures.

The polarization–electric field (*P*–*E*) hysteresis curve appears as a parallelogram loop, providing evidence of ferroelectricity in Phase I (Fig. 3a and S4†). The *P*–*E* curve changes abruptly to double hysteresis loops in a narrow temperature range just above *T*_c_. The corresponding current density (*J*) *versus E* curve (Fig. 3b) changes so that each pair of peaks is further split into doublets. The coercive field, which decreases with temperature in ferroelectric Phase I, is continuously converted at *T*_c_ into the switching field of the double loops (Fig. 3c). Such behavior can be explained by a reversible field-induced paraelectric-to-ferroelectric phase transitions as a characteristic near the strong first-order ferroelectric transition, as previously observed in BaTiO_3_.^35^

#### H55dmbp-Hba

The relative permittivity *ε*_r_ of H55dmbp-Hba obeys the Curie–Weiss law in Phase II and it displays a single sharp maximum, characteristic of a ferroelectric phase transition (Fig. 2b). Compared with an earlier report,^34^ in our reexamination, the peak value of *ε*_r_ was significantly improved to a value of 13 000. H55dmbp-Hba, as well as H55dmbp-Hia and H22bpy-Hia, exhibited a huge Curie–Weiss constant (Table 1), which is about two orders of magnitude larger than *T*_c_. The Curie point (*T*_c_ = 259 K) of H55dmbp-Hba was 9 K lower than that of H55dmbp-Hia (268 K). The phase transition is first order for H55dmbp-Hia and nearly second order for the H55dmbp-Hba crystal, considering the differences between *θ* and *T*_c_ (17 K and <1 K, respectively). This explains why the permittivity showed more-pronounced divergent behavior (a peak value of 13 000) in the latter crystal.

The presence of ferroelectricity was confirmed by the change in the *P*–*E* hysteresis curve from a straight line, through an S-shaped line, to a parallelogram-like loop in Phase I (Fig. S5†). The magnitude of the remanent polarization tended to depend on the specimen, due to differences in crystal quality. By careful selection of regularly shaped crystals, the best performance that we achieved in our reexamination (3.6 μC cm^−2^; Fig. 4) was about three times that in the original report.

#### Effects of deuteration

For all the compounds, deuterium substitution of the hydrogen bonds shifts the permittivity anomalies at *T*_c_ toward higher temperatures by Δ*T*_c_ ≈ 30–70 K, with a small change in the overall *ε*_r_–*T* curvature. The magnitude of polarization was also enlarged on deuteration (Fig. 3c, and 4b). The maximum spontaneous polarization of D22bpy-Dia was as high as 5 μC cm^−2^. Table 1 summarizes the dielectric properties of several supramolecular ferroelectric single crystals, including the reported ones. The isotope effect-induced increases in *T*_c_ and *P*_r_ are typical behaviors for proton transfers within hydrogen bonds. Reflecting the strengthened polarizations, the Curie–Weiss constant *C* also increases with deuteration. Note that the only exception was H55dmbp-Hba, which exhibited a reduction in *C*. A plausible reason for this is that the temperature range for the proper paraelectric Phase II of the D55dmbp-Dba salt is too narrow to permit analysis by the Curie–Weiss law. In this case, the constant *C* was, for convenience, derived from the permittivity of the (nonauthentic paraelectric) Phase III and was, consequently, inappropriate for comparison. The deuterated single crystals are sufficiently chemically robust against reverse substitution under ambient conditions to maintain their phase-transition temperatures for at least five years after their preparation.

#### Theoretical polarizations

Recent quantum simulations of electric polarizations based on Berry phase theory quantitatively explain the experimental polarizations of fully polarized organic ferroelectrics and antiferroelectrics, especially such prototropic compounds as croconic acid or squaric acid.^25,36–38^ Starting from the Phase I crystal structures, the electronic-structure calculations include the energetic relaxation of the locations of hydrogen atoms, which are adjusted to the rational bond geometry (O–H: 1.03–1.05 Å; N–H: 1.09–1.10 Å) of the hydrogen bonds. The theoretical polarizations are given together with their direction components and are compared with the experimental results in Table 1. The D22bpy-Dia crystal exhibited a moderately large maximum *P*_r_ of about 5 μC cm^−2^. On the other hand, the simulation gave a spontaneous polarization of 8.21 μC cm^−2^ with direction components of (*P*_*a*_, *P*_*b*′_, *P*_c*_) = (1.77, 1.72, 7.83) μC cm^−2^ when the *b*′-direction is taken as perpendicular to the *a* and c* axes (Fig. S6†). The calculated polarization vectors are approximately parallel to the hydrogen bonds, as shown by the thick arrows in Fig. 1. The direction component of 7.18 μC cm^−2^ along the electric-field direction [*E*_⊥_ (101) plane] well explains the observed value.

A similar simulation of H55dmbp-Hba gave a spontaneous polarization of 6.73 μC cm^−2^ with direction components of (*P*_*a*_, *P*_*b*′_, *P*_c*_) = (−3.49, −0.11, −5.75) μC cm^−2^. The direction component of 6.39 μC cm^−2^ along the electric-field direction [*E*_⊥_ (101) plane] also explains the observed value.

### Phase-transition sequences

**Fig. 5:**
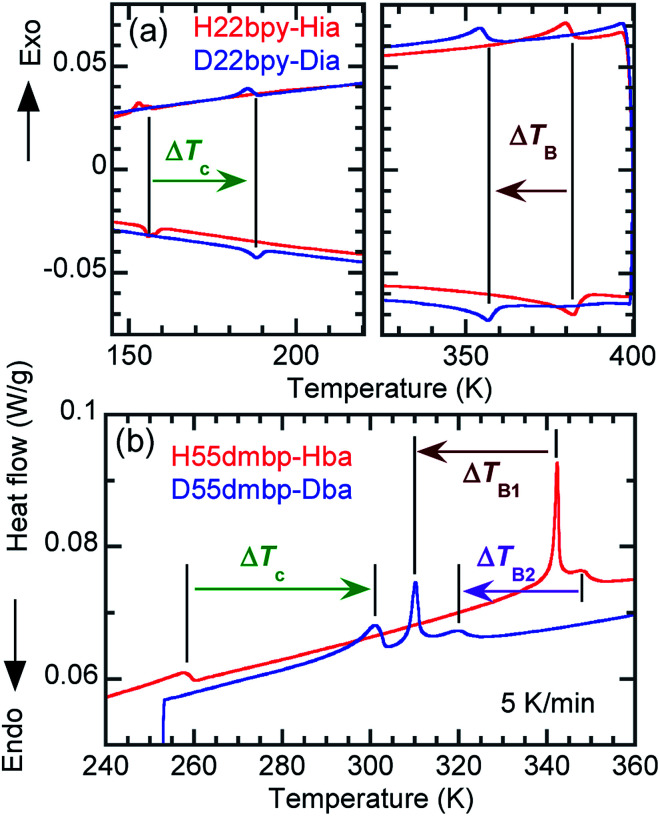
Thermal properties and their deuteration effects. Heat-flow profiles in differential scanning calorimetry measured at a rate of 5 K min^−1^ (a) H55dmbp-Hba and D55dmbp-Dba. (b) H22bpy-Hia and D22bpy-Dia.

An alternative method for identifying phase transitions is by means of the heat-flow profiles determined by high-sensitivity differential scanning calorimetry (DSC). H22bpy-Hia and deuterated D22bpy-Dia showed pairs of exothermal and endothermal peaks at *T*_c_, and their isotope shift was in agreement with the dielectric measurements (Fig. 5a and S7†). These faint peaks due to the small entropy changes suggested that the phase transitions are not the order-disorder type. In contrast, there were no detectable anomalies at *T*_A_ between phases IC and II, probably because the entropy change is too small, and this leaves the possibility of some crossover phenomenon. H55dmbp-Hba, H55dmbp-Hia, and their deuterated salts also exhibited tiny peaks or kink-like anomalies at their Curie points (Fig. 5b and S7f†).

The emergence of Phase III at temperatures far above room temperature is identified by an additional pair of exothermal and endothermal peaks for the H22bpy-Hia and deuterated D22bpy-Dia salts. The reversible phase transition at *T*_B_ = 357 K is also related to a faint kink in the intrastack permittivity (shown by an arrow in Fig. S2†) for the latter crystal. An exotic feature is its downward shift of as much as 25 K upon deuterium substitution of the hydrogen bonds. In sharp contrast, full deuteration of the base (22bpy-*d*_8_) except for the hydrogen-bonding acidic protons has little effect on the phase-transition temperatures (Fig. S7c†). These observations show that hydrogen bonds play crucial roles on both the low- and high-temperature phase transitions.

**Fig. 6:**
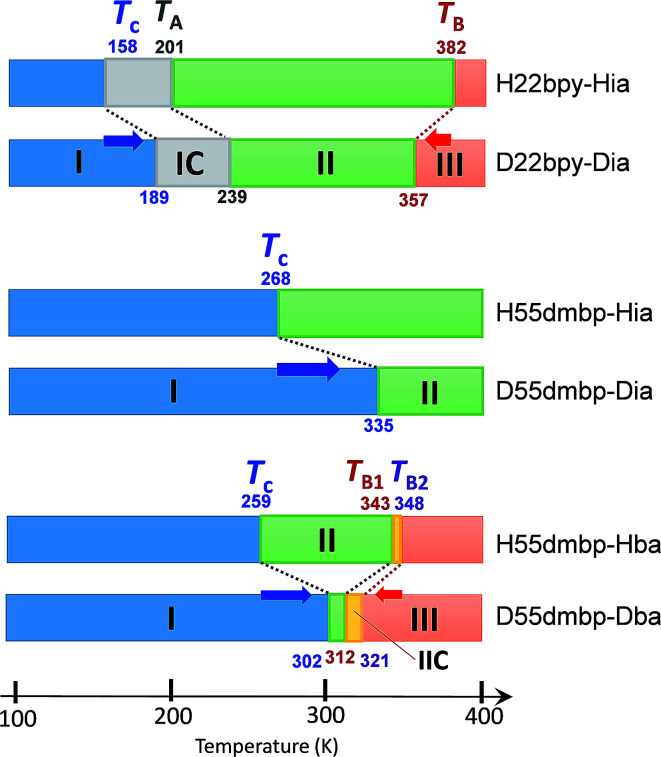
Phase-transition sequences of H22bpy-Hia, H55dmbp-Hba, H55dmbp-Hia, and their deuterated salts. The numbers represent the phase-transition temperatures in Kelvin.

A transition to Phase III likewise appears for the H55dmbp-Hba and deuterated D55dmbp-Dba (Fig. 5b), but is absent in the isomorphous compounds H55dmbp-Hia and D55dmbp-Dia below the thermal-stability limit (Fig. S7e†). Note that the anomaly consists of two phase transitions for H55dmbp-Hba. The sharp peak at *T*_B1_ = 343 K is accompanied by the small and blunt anomaly at *T*_B2_ = 348 K, suggesting that an additional intermediate phase (denoted as IIC) is present between Phases II and III. Deuterium substitution significantly decreases *T*_B1_ and *T*_B2_ by 31 and 28 K, respectively. This inverse isotope effect, together with the normal isotope effect on *T*_c_, dramatically collapses the temperature window of Phase II from 84 to 10 K. On the basis of the electrical and thermal properties, the phase-transition sequences are summarized in Fig. 6.

### Thermal structural changes

#### Lattice and global symmetry

To explore the structural origins of the dielectric properties and the isotope effects, the crystal structures at room temperature were determined for H22bpy-Hia (1h), H22bpy-*d*_8_-Hia (1h′), D22bpy-Dia (1d), D55dmbp-Dia (2d), H55dmbp-Hba (3h), and D55dmbp-Dba (3d) crystals; that of H55dmbp-Hia (2h) has been reported previously.^28^ Table S1† summarizes the crystal data, experimental details, and bond geometries around the hydrogen bonds. Deuteration of the hydrogen bonds in the H22bpy-Hia and H55dmbp-Hba crystals is accompanied by a slight expansion of the unit-cell parameters *a* (+0.29%, +0.14%, respectively) and *c* (+0.12%, +0.14%), and a contraction of *b* (−0.27%, −0.29%).

**Fig. 7:**
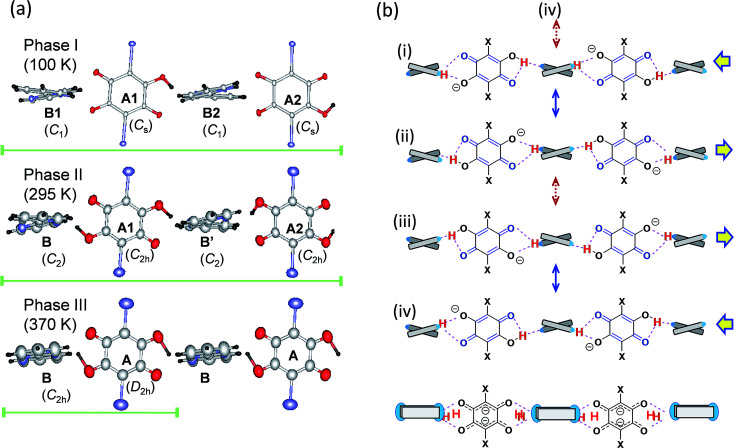
Structural changes in the hydrogen-bonded chains viewed along the longitudinal axis of a twisting base molecule. (a) Molecular arrangements in Phase I (*T* = 100 K), Phase II (295 K), and Phase III (370 K) of a D22bpy-Dia crystal drawn with thermal ellipsoids at the 50% probability level. The bars show the periodicity of the chain. (b) Resonance schemes in the higher-temperature phase structures. Solid double-headed arrows indicate intermixing of two of the contributing structures, describing the disorder in Phase II, whereas the bottom scheme represents the resonance hybrid in the Phase III structures formed by intermixing of all four contributing structures, as indicated by the dotted and solid arrows.

**Fig. 8:**
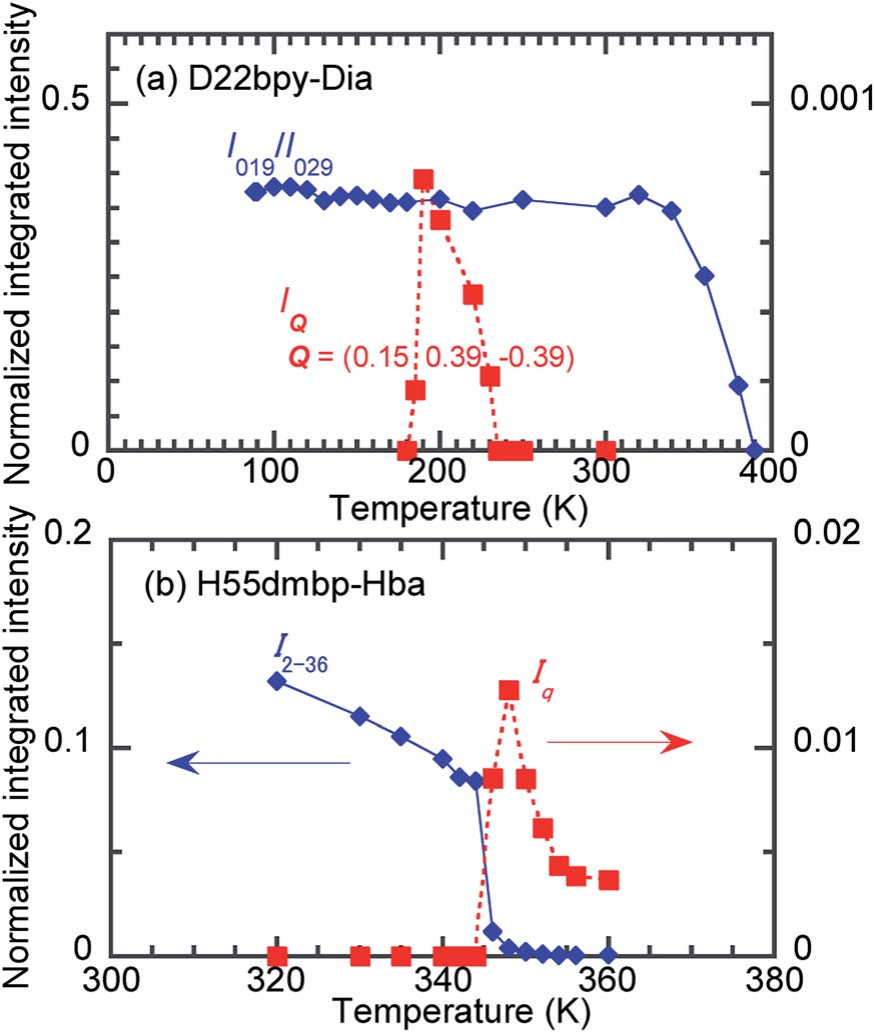
Temperature-dependent integrated intensities of the X-ray diffractions arising from the formation of the doubled (blue) and incommensurate (red) lattice periodicities of the (a) D22bpy-Dia (1d) and (b) H55dmbp-Hba (3h) crystals.

The thermal structural changes of the D22bpy-Dia and H55dmbp-Hba salts were examined. Under ambient conditions, both crystals adopt the Phase II form, which consists of two formula units in a triclinic unit cell. The transition from Phase I to Phase II adds inversion symmetry to each Hxa^−^ molecule, and can thus be of a true ferroelectric type. Note that the D22bpy and H55dmbp molecules remain twisted in these phases. For both compounds, the high-temperature Phase III form retains a triclinic *P*1̄ symmetry, but it consists of only one formula unit in a halved unit-cell volume. Halving of the unit-cell parameter *b* in Phase III is demonstrated by the disappearance of *k* = odd reflections (Fig. 8). The D22bpy and H55dmbp molecules also acquire an inversion symmetry that necessitates that they have exactly coplanar conformations. Fig. 7a illustrates the structural changes in the supramolecular chain from Phase I to Phase III.

In the temperature range for Phase IC, the D22bpy-Dia crystal shows X-ray satellite reflections around each Bragg reflection, suggesting the formation of a superlattice with noninteger periodicity, as shown by Fig. 8a, and S8a.† Therefore, Phase IC just above *T*_c_ is as an ‘incommensurately’ modulated structure. Some modulated structures exist in the very narrow temperature range of Phase IIC (Fig. 8b, and S8b†), although a confirmation of this fact is beyond the scope of this paper.

#### Molecular geometry

A key structure–property relationship concerns the degree of proton transfer and ordering, which determines the lattice symmetry as well as the polarity of the supramolecular chains. Chart 1 schematically classifies the crystal forms into four types: (A) the neutral adduct bpy-H_2_xa, (B) the monovalent Hbpy^+^-Hxa^−^ salt with ordered protons, (C) monovalent salts with disordered (or centered) protons, and (D) divalent H_2_bpy^2+^-xa^2−^ salts. The monovalent form B has bistable polarities on the chains, as required for ferro- and antiferroelectricity, whereas the others forms are nonpolar. According to the X-ray structural analysis, only Phase I satisfies this structural requirement, as expected.

Unfortunately, accurate locations of hydrogen atoms are difficult to determine by X-ray analysis, especially for the disordered case (form C), because the crystals contain heavy bromine or iodine atoms with large scattering factors. A clue to their locations can be obtained from local molecular geometries, which are closely interrelated to the local degrees of proton transfer between the neutral N⋯H–O and ionic N–H^+^⋯O^−^ forms. This analysis has been previously applied to evaluate the ferroelectric phase transition in the H55dmbp-Hia crystal.^28^

The molecular structures of neutral H_2_xa adopt a planar and typical *para*-quinoid form that corresponds to the point-group symmetry *C*_2h_. Their symmetry is lowered to a point-group symmetry of *C*_s_ upon deprotonation to the monovalent Hxa^−^ anions; the C–O^−^ bond with length of 1.25–1.26 Å is substantially shorter than that of the corresponding C–OH bond (bond length 1.32–1.33 Å) with a corresponding lengthening of the adjacent C=C bond. The change in the geometry of the base molecules upon protonation is most obvious in the pyridine ring C=N–C angle, which is 116–117° for the neutral ring and widens to 121–123° for the protonated ring. Therefore, the degrees of proton transfer can be evaluated by plotting the change in the C=N–C angle (*δ*) against the C–O bond length (*d*_C–O_) (Fig. 6b) relative to the shaded boxes *N* and *I*, which denote the standard geometries of the neutral N⋯H–O and ionic N–H^+^⋯O^−^ forms, respectively.

In each supramolecular chain of the ordered Phase I structures, four crystallographically independent hydrogen bonds form a repeating unit, as exemplified by the chemical structure (i) and the corresponding drawings in Fig. 7. Actually, the diagnostic diagram (Fig. S9†) distinguishes two neutral N⋯H–O and two ionic N–H^+^⋯O^−^ bonds as the ordered monovalent state of the protons. The phase transition to a paraelectric form (Phase II) halves the asymmetric unit, and then reduces the number of independent hydrogen bonds to two. Each local bond geometry is dynamically averaged between the N⋯H–O and N–H^+^⋯O^−^ forms, indicating a disorder of the protons in Phase II. Fig. 7b shows the resonance scheme for this disordered structure: the solid double-headed arrows represent intermixes of two contributing structures so that the resonance hybrids (i) ↔ (ii) and (iii) ↔ (iv) preserve the same *para*-quinoid geometry with a point-group symmetry of *C*_2h_. Note that the molecules A1 and A2 are symmetric but crystallographically independent of each other, whereas molecules B and B′ are related to one another by inversion symmetry. This phase transition picture is very similar to the hydrogen dynamics found by the ^2^H magic-angle spinning NMR and ^14^N nuclear quadrupole resonance studies made on the chlorine-substituted H55dmbp-Hca,^39,40^ which is comparable to H55dmbp-Hba salt in both the O⋯N bond lengths and the phase transition temperatures.

In the highest-temperature Phase III, the volumes of the unit cell and asymmetric unit are further halved and contain only one crystallographically independent hydrogen bond. Addition of an inversion symmetry to the base increases the degree of configurational disorder. The additional intermixing process indicated by the dotted double-headed arrows in Fig. 7b involves an orientational switching of the *para*-quinoid structure. The bottom scheme represents the resonance hybrid for the Phase III structures formed from all four contributing structures. The anilate ion in the hybrid resonance approximately exhibits a molecular structure of point-group symmetry *D*_2h_, and its divalent character is consistent with the local hydrogen-bond geometry, shown as increasing ionicity in the diagram (Fig. S9†).

Besides the hydrogen-bond geometry, the molecular flexibility of the base is a key characteristic of the high-temperature phase transition, above which temperature the twisted conformation becomes coplanar with the emergence of an inversion symmetry. A fundamental concern regarding neutral and protonated 22bpy molecules is the relative geometric stability of the *cis* and *trans* forms and of the coplanar and twisted forms. For the monoprotonated H-22bpy^+^ cation, the *cis* form is relatively stable due to presence of the intramolecular N–H^+^⋯N bond, and the *cis*–*trans* interconversion energy has been estimated to be 14 kJ mol^−1^.^41^ Actually, both *cis* and *trans* forms exist in the three polymorphs of the H66dmbp-Hca salt. The ferroelectric D22bpy-Dia and H55dmbp-Hba salts were shown to exist in a twisted *trans* form at both phases I and II. At room temperature, the corresponding NCCN torsion angles are 16.7° and 12.6°, respectively, which are less than that of the H55dmbp-Hia salt (16.9°). The torsion angles increase on lowering the temperature below *T*_c_; 17.6 and 17.9° at 100 K for D22bpy-Dia and 13.5 and 14.8° at 173 K for H55dmbp-Hba. Therefore, the presence of a smaller twisting angle might explain the easier transformation into coplanar conformation with the emergence of Phase III. To summarize the structural transformations discussed above, the phase sequence I → II → III successively involves symmetry elements on both molecules as described in terms of point-group symmetry as *C*_1_ → *C*_2_ → *C*_2h_ for Hbpy^+^ and *C*_s_ → *C*_2h_ → *D*_2h_ for Hxa^−^.

### Hydrogen-bond geometry and deuteration effects

KH_2_PO_4_ (KDP)-type ferroelectrics exhibit strong correlations between some of their macroscopic properties and their hydrogen-bond lengths. For instance, the application of a hydrostatic pressure shrinks the separation of the two equilibrium proton positions and weakens the potential-energy barrier between the two minima. The Curie point is decreased toward zero temperature during destabilization of the ferroelectric state until the protons occupy a single minimum at the midpoint of the bond.^42^ On the other hand, the substitution of protons for deuterons significantly diminishes quantum-mechanical tunneling and favors off-center locations due to a distinct zero-point motion. This is also accompanied by a feedback effect of hydrogen-bond elongation known as the Ubbelohde effect,^43^ and the combined effects create a huge isotope effect manifested as significant increases in both the Curie point and hydrogen-bond length. For the series of supramolecular ferroelectrics, both the *T*_c_ and *P*_s_ increase monotonously with increasing hydrogen-bond length.^30^ Actually, deuteration of the hydrogen bonds elongates the nearest O⋯N bond length *d*_O⋯N(1)_ by 0.03–0.05 Å (Table S1†); the observed positive *T*_c_ can then be understood by analogy with that of KDP. Note that full deuteration of pyridine C–H sites does not produce this geometric effect, in agreement with the unchanged *T*_c_. On the other hand, other structural mechanisms should be considered for the negative shift in the transition point at higher temperatures.

Among hydrogen-bonded ferroelectrics, Rochelle salt (potassium sodium tartrate tetrahydrate) exhibits an exceptional isotope effect, which has been long known since Valasek's findings regarding the reentrant phase transition^44^ and the inverse isotope effect,^45^ following his initial discovery of ferroelectricity in 1920.^46^ A novel feature of Rochelle salt is its intermediate ferroelectric phase that penetrates through a chiral paraelectric ground state in a narrow temperature range (255–297 K). Deuterium substitution raises the upper Curie point by 11 K, whereas an inverse effect with a shift of only 5 K emerges for the lower (*i.e.*, reentrant) Curie point. These behaviors are consistent with a model of the KDP family in which deuteration energetically stabilizes the ferroelectric phase relative to the paraelectric one. Compared with the observations for Rochelle salt, deuterium substitution of our two supramolecular ferroelectrics has an opposite but much stronger influence on the thermal stability of the intermediate phase. The other distinct feature is that the present ferroelectrics do not show a reentrant phase.

**Fig. 9:**
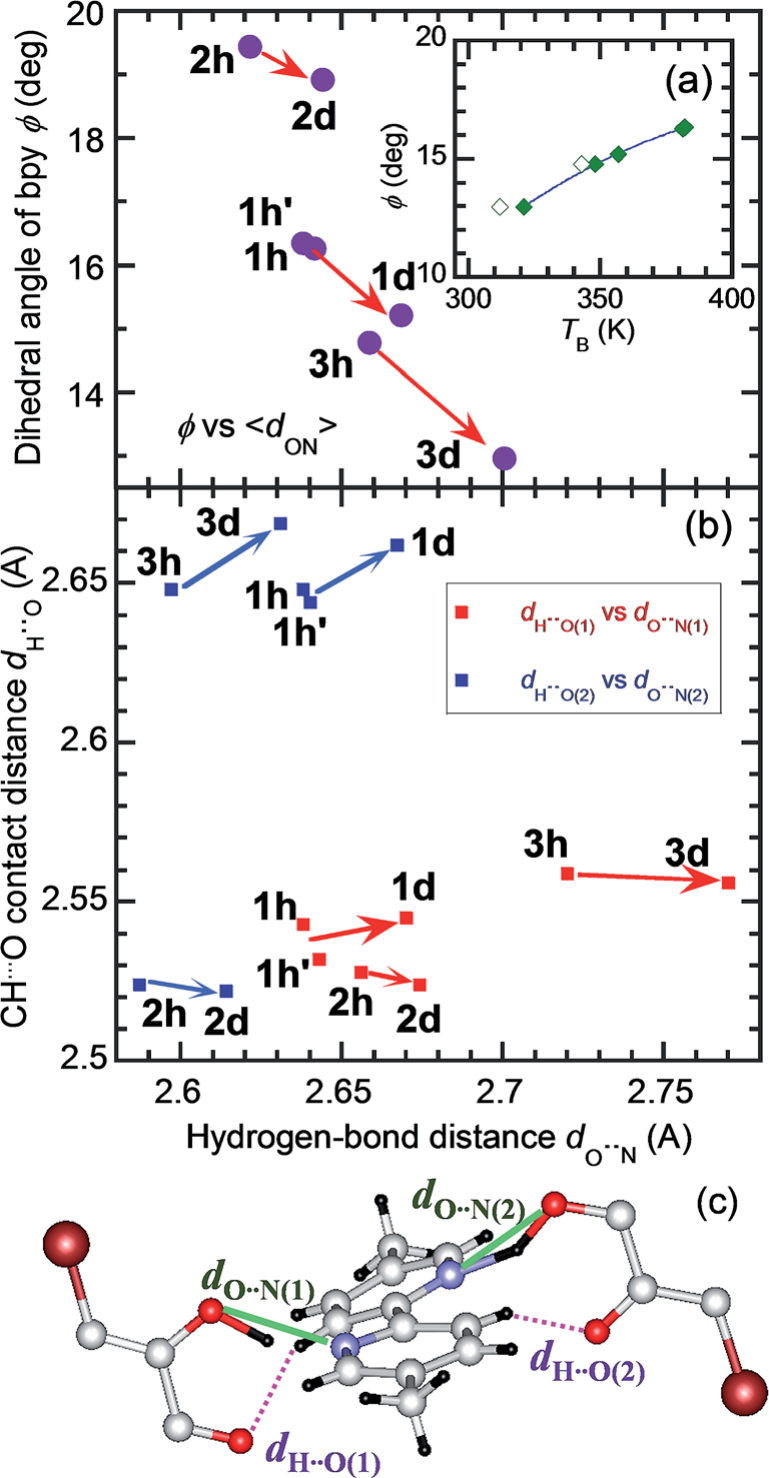
Correlation between the hydrogen-bonding geometry and twisting deformation of bipyridine molecules in the H22bpy-Hia (1h), H22bpy-*d*_8_-Hia (1h′), D22bpy-Dia (1d), H55dmbp-Hia (2h), D55dmbp-Dia (2d), H55dmbp-Hba (3h), and D55dmbp-Dba (3d) crystals. The arrows represent the effects of the deuterated hydrogen bonds. (a) The twisting angles *φ versus* averaged hydrogen-bond lengths 〈*d*_O⋯N_〉. (Inset) Correlation between the *φ* and *T*_B_ (or *T*_B1_; open squares). (b) Plot of the CH⋯O distance *d*_H⋯O(1)_ [or *d*_H⋯O(2)_] *versus d*_O⋯N(1)_ [or *d*_O⋯N(2)_]. See panel (c) for the notation of the structural parameters.

The strong temperature shifts suggest that hydrogen bonds play crucial roles in the microscopic mechanisms of the high-temperature phase transition and the low-temperature ferroelectric transition. These phase transitions are commonly accompanied by increased degrees of disorder; the number of configurations involved in the resonance hybrid increases with temperature from one in Phase I through two in Phase II to four in Phase III. On the other hand, the microscopic features that are exclusively present in the high-temperature phase-transition (coupling between the hydrogen-bond geometry and the twisting deformation of the bipyridine) should provide a clue for identifying the origins of the exotic isotope effect. The inset to Fig. 9a shows the systematic increase in the phase-transition temperature *T*_B_ (or *T*_B1_ and *T*_B2_) with an increase in the twisting angle *φ*, defined as the dihedral angle between the two pyridyl rings at room temperature. The extrapolation of this curve also explains the nominally high *T*_B_ concealed beyond the onset temperature of decomposition (∼418 K) for H55dmbp-Hia and D55dmbp-Dia with larger values of *φ* (19.5° and 18.9°, respectively). These behaviors suggest that *φ* can be used as the corresponding order parameter, so that a smaller value of *φ* permits easier transformation to the coplanar conformation in Phase III near to or above room temperature.

The origin of the inverse isotope effect is relevant to how the geometry of the hydrogen bonds is interrelated to the twisting deformation. Fig. 9c depicts the crystallographically independent unit in the Phase II structure to highlight the intermolecular contacts between the hydrogen-bonded molecules. Each pyridyl ring is involved in hydrogen bonding of a bifurcated type with two oxygen atoms; the hydroxyl oxygen atom with the shortest distance *d*_O⋯N(1)_ (or *d*_O⋯N(2)_) and the carboxyl oxygen atom in the next-nearest neighbor. Besides such moderately strong hydrogen bonding, this molecular pair exhibits weak CH⋯O hydrogen bonding, which appears as the only intermolecular contact with a shorter distance than the sum of the corresponding van der Waals radii. The H⋯O distance *d*_H⋯O(1)_ [or *d*_H⋯O(2)_; Fig. 9c] between the carbonyl oxygen atom and the remainder of the pyridyl ring ranges from 2.52 to 2.67 Å. Because both pyridyl rings interact with each Hxa^−^ ion in the neighborhood, their dihedral angle is determined by these bond geometries. For all three compounds examined, deuteration similarly decreases the twisting angles *φ* and increases the average hydrogen bond length 〈*d*_O⋯N_〉, calculated by averaging over all the crystallographically independent sites at room temperature [*e.g.*, *d*_O⋯N(1)_ and *d*_O⋯N(2)_ in Fig. 9a]. On the other hand, deuteration has little effect on *d*_H⋯O(1)_ and *d*_H⋯O(2)_ (Fig. 9b). These observations show that the geometric isotope effects adjust the molecular conformation of the flexible bipyridine units to minimize the change in adjacent CH⋯O interactions.

Few other deuterium-bonded crystals exhibit a relatively large downward shift in the phase-transition temperature. Note that these inverse isotope effects are similarly explained by geometric isotope effects on molecular dynamics, although the details of the induced structural changes vary with the material. For example, K_3_H(SO_4_)_2_ crystals exhibit a shift of −9 K for the phase transition from the ferroelastic to superionic state.^47^ A larger shift of −33 K has recently been reported for the antiferroelectric crystal of imidazolium hydrogen terephthalate.^11^ This observation was explained in terms of the host–guest framework, in which a deuteration-induced geometric change suppresses freezing of the libration of the guest molecules in the loosened packing space.

## Experimental

Commercially available H_2_ba, 22bpy, and 22bpy-*d*_8_ were repeatedly purified by vacuum sublimation. H_2_ia, prepared from *p*-iodanil by the reported method,^48^ was purified twice by gradient sublimation in vacuum until its purity became satisfactory, as assessed by elementary analysis and mass spectrometry. Reddish brown plates of the H55dmbp-Hba and H55dmbp-Hia salts, as well as dark-brown plates of the H22bpy-Hia salt, were grown by slow evaporation of methanolic solutions at room temperature. Crystal growth of H55dmbp-Hba was performed by seeding with crystals to avoid the preferential formation of a fibrous polymorph of unknown crystal structure.

Deuterium substitution of the hydrogen bonds was achieved by the slow evaporation of a mixed solution in 99.5%-deuterated CH_3_OD (Aldrich). The degrees of deuteration of D55dmbp-Dba (94%), D55dmbp-Dia (93%), and D22bpy-Dia (85%) were determined by infrared vibrational spectroscopy in KBr disks. The amount of residual nondeuterated species was estimated from the reduction in the band intensity for the nondeuterated acid at 970 cm^−1^ (H_2_ba) or 958 cm^−1^ (H_2_ia) by using the band at 1040 cm^−1^ (55dmbp) or 1001 cm^−1^ (22bpy) as an internal relative standard.

The permittivity was measured with an LCR meter (4284A Precision; Agilent Technologies, Santa Clara, CA) by using single crystals with painted silver or carbon-paste electrodes. The *P*–*E* hysteresis curves were measured on a ferroelectrics evaluation system (FCE-1; Toyo Corp., Kariya) consisting of a current/charge–voltage converter (Model 6252), an arbitrary waveform generator (Biomation 2414B), an analogue-to-digital converter (WaveBook 516), and a voltage amplifier (HVA4321; NF Corp.). The measurements at room temperature were performed with a high-voltage triangular wave field and various alternating frequencies.

The high-sensitivity thermal analysis was performed by using a differential scanning calorimeter (DSC7000X; Hitachi High-Technologies Corp., Tokyo). The crystals (10–20 mg) were crushed and encapsulated in an aluminum pan and cooled (or heated) at a rate of 5 K min^−1^ under nitrogen flowing at 60 mL min^−1^. The temperature was calibrated by using the melting point of indium (429.8 K).

Collection of the X-ray diffraction data at room temperature and the assignment of the crystallographic axes of the bulk single crystals were performed by using graphite-monochromated MoKα radiation (*λ* = 0.71073 Å) and a four-circle diffractometer equipped with a hybrid pixel detector (Rigaku AFC10 with PILATUS200K). The intensity data were analyzed by means of the Crystal Structure crystallographic software packages (Molecular Structure Corp. and Rigaku Corp.). The final refinements of the nonhydrogen atoms were performed with anisotropic thermal factors. The hydrogen-bonded hydrogen atoms were found by means of differential Fourier synthesis and refined isotropically, and the other hydrogen atoms were calculated in their ideal geometrical positions. The same setup was employed for the analysis of the H55dmbp-Hba crystal at 173 K in a stream of cooled nitrogen gas.

The temperature-variable diffraction data were collected by using synchrotron X-rays, except for the H55dmbp-Hba crystal at 173 K. The diffraction experiments were conducted on Rigaku DSC diffractometers equipped with an imaging plate detector installed in beamlines BL-8A and BL-8B of the Photon Factory of the High Energy Accelerator Research Organization (KEK). The X-ray (*λ* = 0.689 or 1.00 Å) beam, monochromatized by using a Si(111) double crystal, was collimated to 0.3 × 0.3 mm by a collimator set immediately upstream of the sample. The reflection intensity data were collected by using the Rapid-AUTO software package (Rigaku Corp.). All the calculations were performed by using the Crystal Structure crystallographic software package.

Electronic polarizations were evaluated by means of the Berry phase approach^49,50^ with the QMAS code^51^ based on the projector augmented-wave method^52^ and the plane–wave basis set. To describe the electronic exchange–correlation energy, the Perdew–Burke–Ernzerhof (PBE) version of the generalized gradient approximation (GGA)^53^ was used. The total polarization was obtained as the sum of the electronic polarization and the ionic polarization; further details are described elsewhere.^54,55^ The target ferroelectric structures (degree of polar distortion *λ* = 1) were constructed from the atomic coordinates of all the nonhydrogen atoms as determined by the experiments. The locations of the hydrogen atoms were computationally relaxed so as to minimize the total energy. The reference paraelectric structures (*λ* = 0) were constructed from averaged molecular structures for *λ* = ±1.

## Conclusions

Supramolecular ferroelectric H22bpy-Hia and H55dmbp-Hba salts show a large relative permittivity (maximum 13 000), a large spontaneous polarization (maximum 5 μC cm^−2^), and a low coercive field ranging from 0.5 to 10 kV cm^−1^. The ferroelectric phase transition at low temperatures is followed by successive phase transitions and exotic isotope effects at higher temperatures. Deuterium substitution over the hydrogen bonds smoothly raises the Curie point in a similar manner to that of KDP, but unexpectedly lowers another phase-transition temperature by a few tens of degrees Kelvin. The cooccurrence of opposite isotope effects reduces the temperature interval of the intermediate paraelectric Phase II of H55dmbp-Hba from 84 to 10 K. This observation is in contrast to effect of deuteration of Rochelle salt, which expands the temperature range for the intermediate ferroelectric phase.

According to temperature-variable X-ray diffraction studies, both phase transitions modify the molecular and hydrogen-bonded geometries, as expected from their significant isotope effects. Both events are usually accompanied by increased degrees of disorder with temperature, as in the case of the KDP family. Nevertheless, the conventional picture for KDP cannot explain the microscopic origins of the exotic isotope effect on the high-temperature phase transitions. The bipyridine molecules exhibit an interplanar twisting, which represents an order parameter relevant to the high-temperature phase transitions. The inverse temperature shift originates from the indirect effect of the hydrogen-bond geometry, which adjusts the molecular conformation of the flexible bipyridine unit so as to minimize the change in adjacent intermolecular CH⋯O interactions.

## Conflicts of interest

There are no conflicts to declare.

## Supplementary Material

RA-009-C9RA06489C-s001

RA-009-C9RA06489C-s002
