# Supplementary material for: Coexistence of normal and inverse deuterium isotope effects in a phase-transition sequence of organic ferroelectrics
Source: RSC Adv. 2019 Dec 2;9(68):39662–73. doi: 10.1039/c9ra06489c (PMC9076125; doi:10.1039/c9ra06489c)
Supplement: RA-009-C9RA06489C-s001 [file RA-009-C9RA06489C-s001.pdf]

Electronic Supplementary Material (ESI) for RSC Advances.
This journal is © The Royal Society of Chemistry 2019

Coexistence of normal and inverse deuterium isotope effects in a phase-transition sequence of organic ferroelectrics

Sachio Horiuchi[1], Shoji Ishibashi[2], Kensuke Kobayashi[3], and, Reiji Kumai[3]*

[1] Electronics and Photonics Research Institute (ESPRIT), National Institute of Advanced Industrial Science and Technology (AIST), Tsukuba 305-8565, Japan

[2] Research Center for Computational Design of Advanced Functional Materials (CD-FMat), National Institute of Advanced Industrial Science and Technology (AIST), Tsukuba, Ibaraki 305-8568, Japan.

[3] Condensed Matter Research Center (CMRC) and Photon Factory, Institute of Materials Structure Science, High Energy Accelerator Research Organization (KEK), Tsukuba, Ibaraki 305-0801, Japan.

Electronic Supplementary Information (ESI)

Experimental details

Electrical Measurements

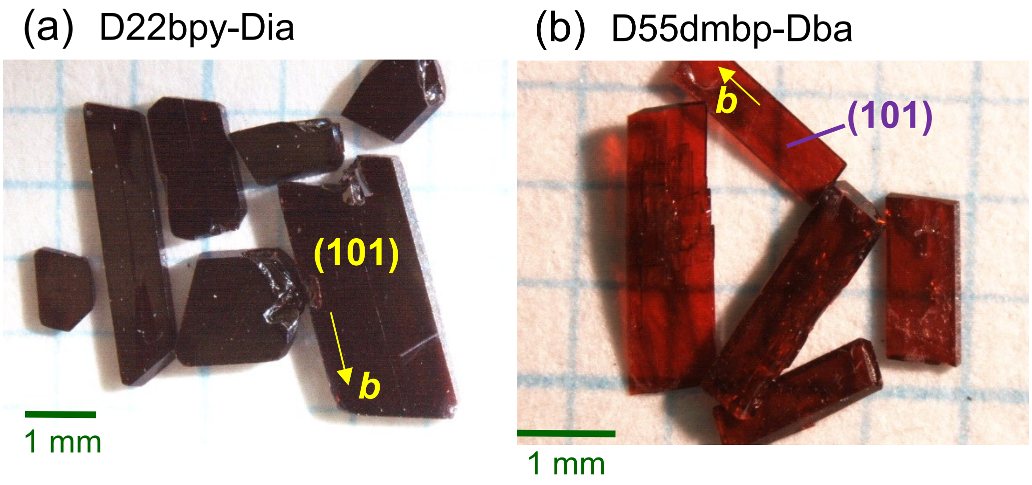

Figure S1. Photographs of single crystals: (a) D22bpy-Dia, (b) D55dmbp-Dba.

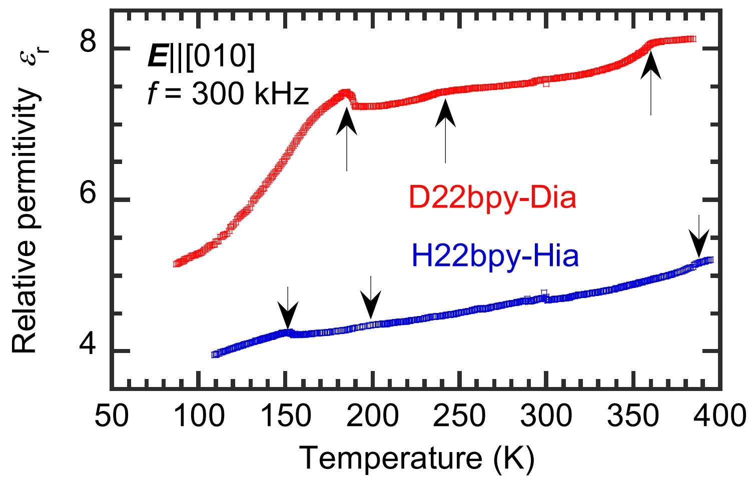

Figure S2. Temperature-dependent *b*-axis permittivity of H22bpy-Hia and D22bpy-Dia single crystals measured with an ac field (300 kHz). The arrows indicate faint anomalies at the phase-transition temperatures.

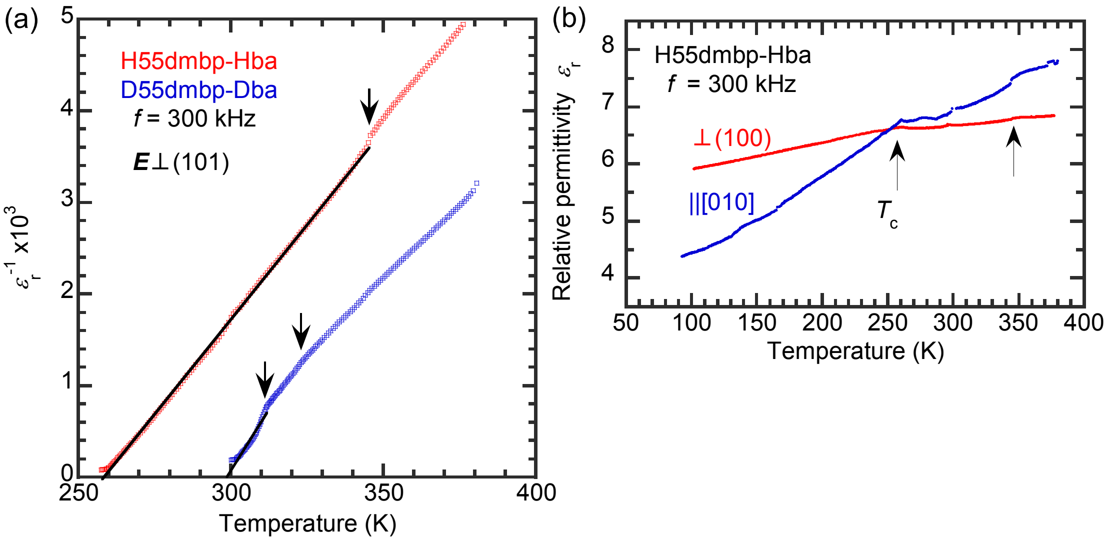

Figure S3. Temperature-dependent dielectric properties of H55dmbp-Hba and D55dmbp-Dba single crystals measured with an ac field (300 kHz). (a) Inverse permittivity ε_r^{-1} measured with an ac field applied normal to the crystal (101) plane. (b) Relative permittivity measured along the interchain directions. The arrows indicate anomalies at the phase-transition temperatures.

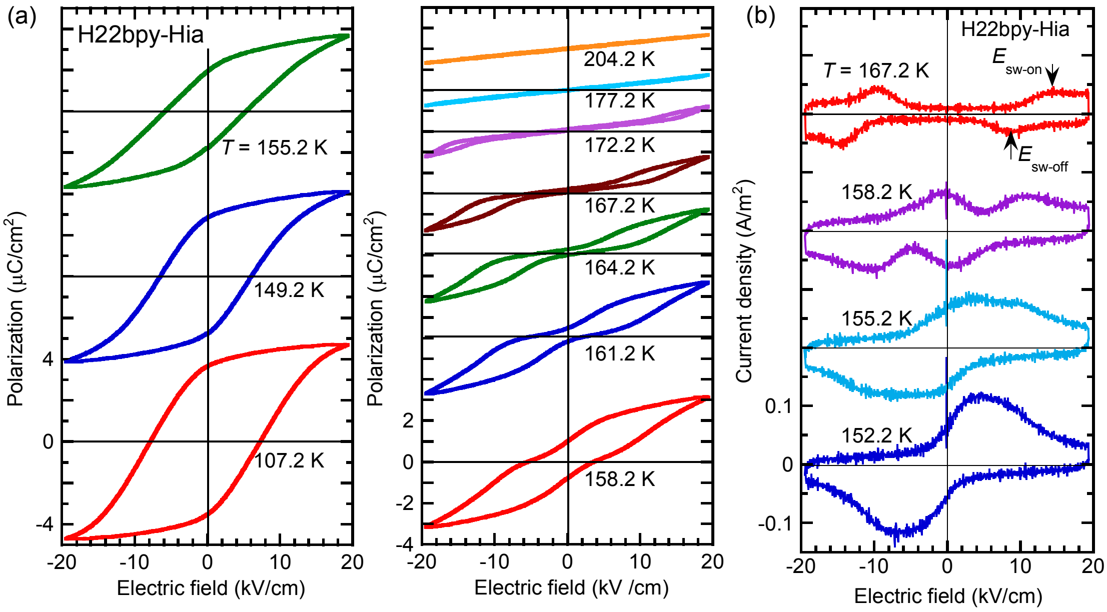

Figure S4. Variations in the polarization-switching properties of a H22bpy-Hia single crystal with temperature. (a) Electric polarization (P) versus electric field (E) hysteresis loops at temperatures below T_c (left) and above T_c (right). (b) Corresponding current density (J) versus E curves. A triangular wave voltage of $f = 0.3$ Hz was applied normal to the crystal (101) plane.

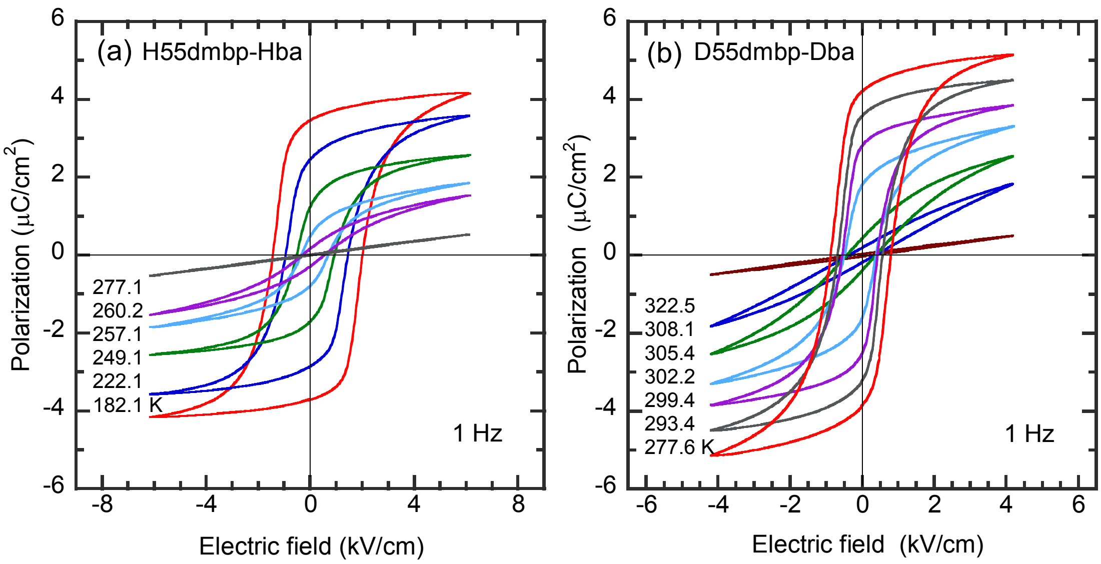

Figure S5. Variation of the *P*–*E* hysteresis loops of (a) H55dmbp-Hba and (b) D55dmbp-Dba single crystals with temperature measured with a triangular waveform voltage (1 Hz) applied normal to the crystal (101) plane.

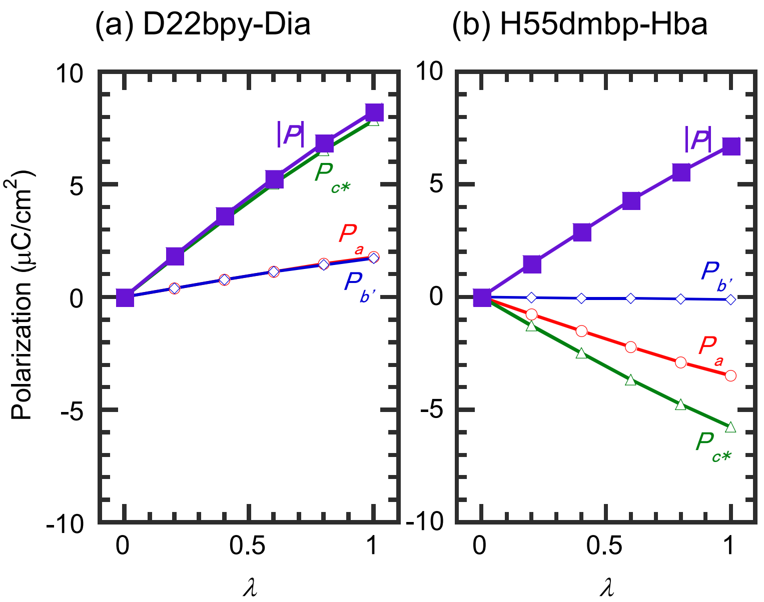

Figure S6. Theoretical spontaneous polarizations as functions of the degree of polar distortion λ on changing from the centrosymmetric reference configuration (hypothetical paraelectric; λ = 0) to a fully polarized (ferroelectric; λ = 1) configuration for (a) D22bpy-Dia and (b) H55dmbp-Hba crystals.

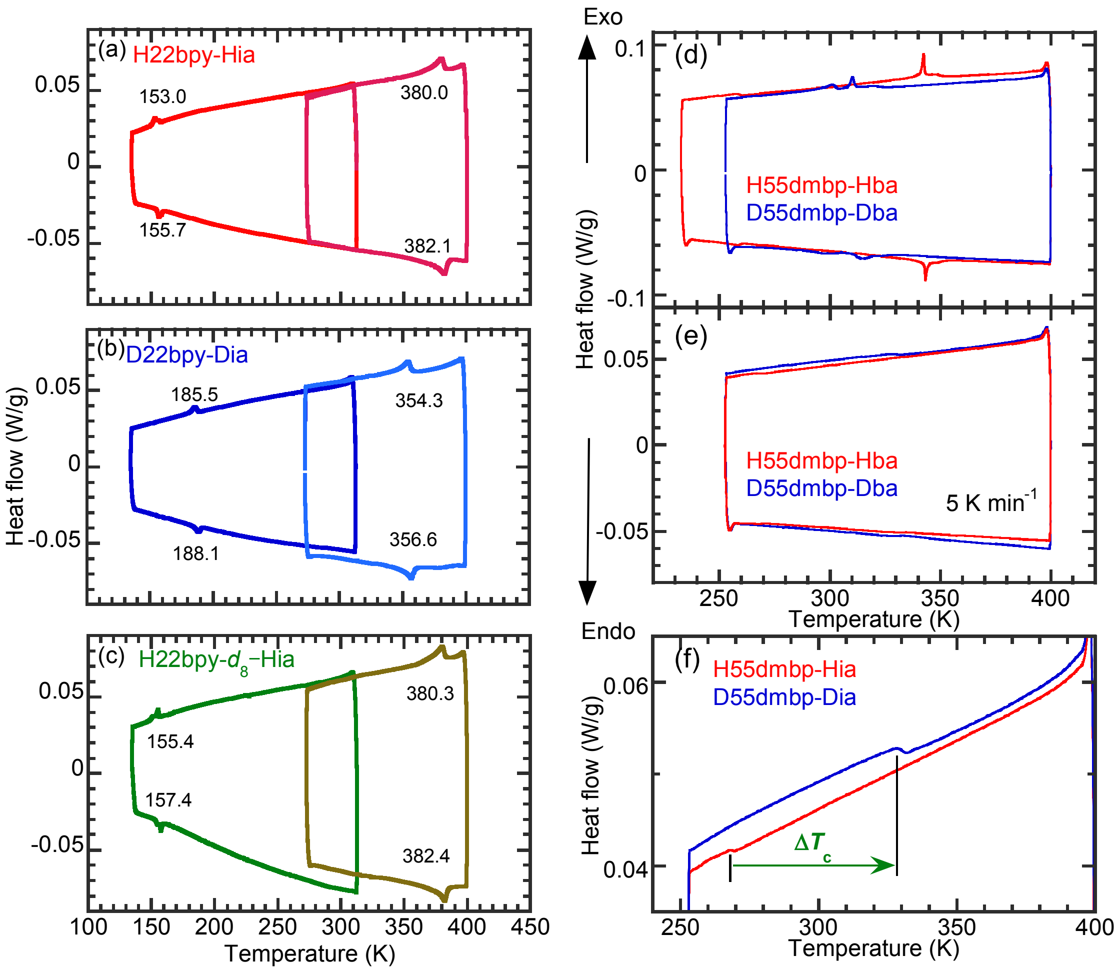

Figure S7. Heat-flow profiles in the differential scanning calorimetry measured at a rate of 5 K·min^{-1}. (a) H22bpy-Hia. (b) D22bpy-Dia. (c) H22bpy-d_8-Hia. (d) H55dmbp-Hba and D55dmbp-Dba. (e) and (f) H55dmbp-Hia and D55dmbp-Dia.

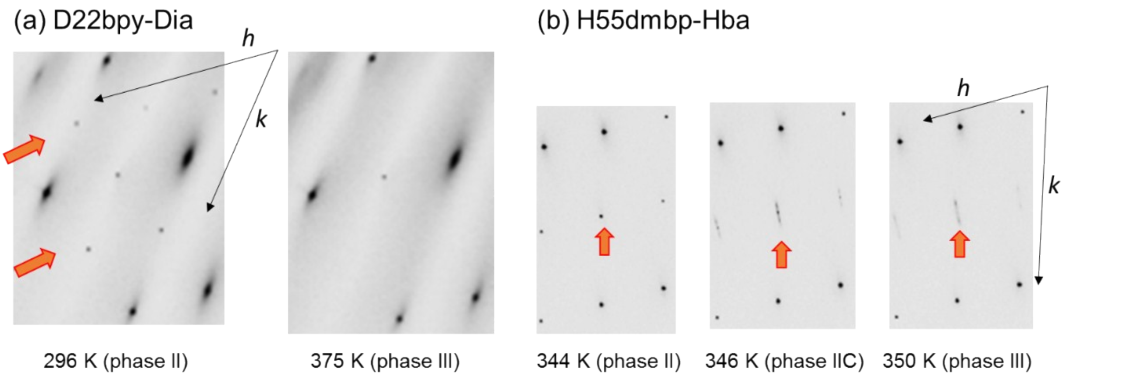

Figure S8. Oscillation photographs recorded on an imaging plate by using synchrotron-radiated X-rays, showing the different structural phases for (a) D22bpy-Dia and (b) H55dmbp-Hba.

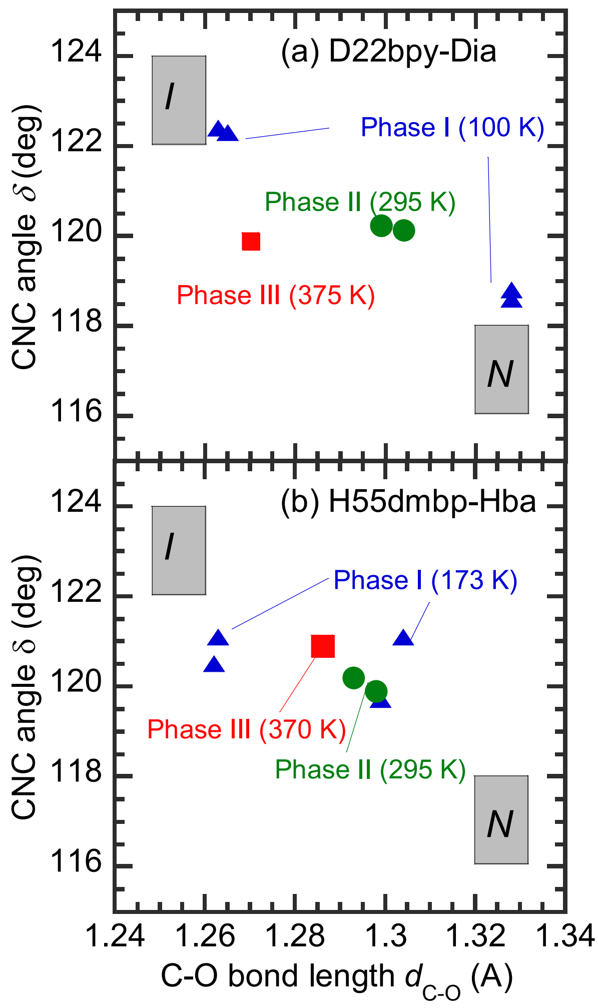

Figure S9. Plot of the C=N−C bond angles δ versus the C−O bond length $d_{C\text{–}O}$ for evaluating the degrees of proton transfer in each hydrogen-bonded site. (a) D22bpy-Dia and (b) H55dmbp-Hba crystals. The shaded boxes *N* and *I* represent the standard geometries of the neutral N···H−O and ionic N−H^+···O^- forms, respectively.

Supplementary Table S1: Crystal data, experimental details, and selected local bond geometries around the hydrogen bonds in single crystals of various supramolecular ferroelectrics at room temperature (295 K)

	H55dmbp-Hba	D55dmbp-Dba	H22bpy-Hia	D22bpy-Dia	H22bpy-d_8-Hia
Chemical formula	$C_{18}H_{14}Br_2N_2O_4$	$C_{18}H_{14}Br_2N_2O_4$	$C_{16}H_{10}I_2N_2O_4$	$C_{16}H_{10}I_2N_2O_4$	$C_{16}H_{10}I_2N_2O_4$
formula weight (*Z*)	482.13 (2)	482.13 (2)	548.07 (2)	548.07 (2)	548.07 (2)
a (Å)	8.812(3)	8.824(2)	8.4976(15)	8.5223(19)	8.4936(15)
b (Å)	9.795(3)	9.767(2)	9.6911(17)	9.665(2)	9.6878(17)
c (Å)	11.993(3)	12.010(3)	11.242(2)	11.255(3)	11.2320(19)
α (deg)	97.0435(19)	97.0167(16)	102.877(3)	102.807(3)	102.855(2)
β (deg)	105.051(4)	104.879(3)	100.665(2)	100.659(2)	100.7131(18)
γ (deg)	115.222(4)	115.300(3)	107.8887(17)	107.876(3)	107.846(3)
V (Å^3)	871.5(5)	872.0(4)	826.2(2)	827.8(3)	824.9(2)
Crystal system	triclinic	triclinic	triclinic	triclinic	triclinic
Space group	*P*-1 (#2)	*P*-1 (#2)	*P*-1 (#2)	*P*-1 (#2)	*P*-1 (#2)
ρ_{calc} (g·cm^{-3})	1.837	1.836	2.203	2.199	2.207
Dimensions (mm)	0.35 × 0.14 × 0.04	0.40 × 0.28 × 0.05	0.28 × 0.28 × 0.16	0.39 × 0.35 × 0.16	0.40 × 0.35 × 0.10
Radiation	MoKα	MoKα	MoKα	MoKα	MoKα
$2\theta_{max}$ (deg)	55	55	55	55	55
R_{int}	0.029	0.031	0.030	0.024	0.043
Reflections used	3977	3983	3754	3762	3761
No. of variables	246	246	226	226	226
R ($2\sigma(I) < I$)	0.0348	0.0338	0.0274	0.0266	0.0303
R_w (All reflections)	0.0999	0.0956	0.0705	0.0660	0.0692
GOF	1.09	1.05	1.07	1.10	1.03
$d_{C-O(1)}$, $d_{C-O(2)}$ (Å)	1,298(3), 1.293(3)	1.289(3), 1.286(3)	1.305(3), 1.296(3)	1.301(3), 1.298(3)	1.300(3), 1.297(3)
∠CNC; δ_1, δ_2 (deg)	119.9(2), 120.2(2)	120.2(2), 120.5(2)	119.4(2), 119.1(2)	119.6(2), 119.1(2)	119.7(2), 119.3(2)
O··N; $d_{O\cdot\cdot N(1)}$, $d_{O\cdot\cdot N(2)}$	2.713(3), 2.592(3)	2.760(2), 2.624(3)	2.631(2), 2.629(3)	2.661(2), 2.659(3)	2.636(2), 2.631(3)
$d'_{O\cdot\cdot N(1)}$, $d'_{O\cdot\cdot N(2)}$ (Å)	2.919(3), 3.046(3)	2.893(3), 3.057(2)	3.013(3), 3.007(2)	3.017(3), 3.009(2)	3.012(3), 3.014(2)
Bpy, dihedral (deg)	14.82(10)	12.97(9)	16.42(10)	15.28(10)	16.35(11)

See Figure S9 for definitions of the chemical-bond parameters $d_{C-O(1)}$, $d_{C-O(2)}$, δ_1, δ_2, $d_{O\cdot\cdot N(1)}$, $d_{O\cdot\cdot N(2)}$, $d'_{O\cdot\cdot N(1)}$, and $d'_{O\cdot\cdot N(2)}$.

Supplementary Table S2: Temperature-dependent crystal data, experimental details, and selected local bond geometries around the hydrogen bonds in single crystals of ferroelectrics

	H55dmbp-Hba	H55dmbp-Hba	D22bpy-Dia	D22bpy-Dia	D22bpy-Dia
Chemical formula	$C_{18}H_{14}Br_2N_2O_4$	$C_{18}H_{14}Br_2N_2O_4$	$C_{16}H_8D_2I_2N_2O_4$	$C_{16}H_8D_2I_2N_2O_4$	$C_{16}H_8D_2I_2N_2O_4$
Formula weight (*Z*)	482.13 (1)	482.13 (2)	550.07 (1)	550.07 (2)	550.07 (2)
T (K)	360	173	375	296	100
a (Å)	4.8779(2)	8.8044(16)	4.8002(3)	8.5359(2)	8.3748(2)
b (Å)	8.1042(3)	9.7839(17)	8.5129(5)	9.6656(2)	9.6561(2)
c (Å)	11.8636(7)	11.882(2)	11.2407(7)	11.2585(7)	11.1482(7)
α (deg)	108.4731(13)	97.226(2)	102.1304(9)	102.8436(7)	102.5857(7)
β (deg)	91.2897(17)	104.520(2)	101.7257(10)	100.6667(7)	100.6515(7)
γ (deg)	98.2241(15)	115.868(3)	104.5226(11)	107.8250(7)	107.6011(7)
V (Å)^3	439.09(4)	858.4(3)	418.54(5)	829.48(6)	807.64(6)
Crystal system	triclinic	triclinic	triclinic	triclinic	triclinic
Space group	*P*-1 (#2)	*P*1 (#1)	*P*-1 (#2)	*P*-1 (#2)	*P*1 (#1)
ρ_{calc} (g·cm^{-3})	1.823	1.842	2.174	2.194	2.254
Dimensions (mm)	0.20 × 0.10 × 0.03	0.27 × 0.21 × 0.06	0.10 × 0.10 × 0.10	0.10 × 0.10 × 0.10	0.10 × 0.10 × 0.10
Radiation	Synchrotron<br>λ = 1.000 Å	MoKα	Synchrotron<br>λ = 0.6875 Å	Synchrotron<br>λ = 0.6875 Å	Synchrotron<br>λ = 0.6869 Å
$2\theta_{max}$ (deg)	90	55	60	70	60
R_{int}	0.029	0.024	0.024	0.016	0.015
Reflections used [$2\sigma(I) < I$]	1210	7435	1798	3916	6647
No. of variables	120	490	130	217	433
R	0.0383	0.0285	0.040	0.0280	0.0160
R_w	0.0940	0.0562	0.1066	0.079	0.0440
GOF	1.079	1.039	1.090	1.044	1.026
$d_{C-O(1)}$, $d_{C-O(2)}$ (Å)	1.286(5), 1.256(3)	1.299(11),1.317(11),<br>1.304(10),1.263(10)<br>1.216(10),1.232(10)<br>1.234(10),1.221(10)	1.270(4),1.259(5)	1.304(3), 1.299(2),<br>1.232(2), 1.232(3)	1.328(5), 1.328(6)<br>1.265(5), 1.263(6)<br>1.238(6), 1.222(6),<br>1.247(5), 1.220(5)
∠CNC; δ_1, δ_2 (deg)	120.9(2)	121.1(7), 121.1(7)<br>119.7(7), 120.5(7)	119.9(3)	120.14(18), 120.24(18)	122.3(5), 118.6(5)<br>122.4(4), 118.8(4)
O··N; $d_{O\cdot\cdot N(1)}$, $d_{O\cdot\cdot N(2)}$ (Å)	2.704(2)	2.730(8), 2.611(10)<br>2.590(11), 2.605(8)	2.830(3)	2.665(2), 2.6731(17)	2.690(2), 2.701(3)<br>2.554(2), 2.601(3)
Bpy, dihedral (deg)		16.7(3)			

See Figure S9 for definitions of chemical-bond parameters, $d_{\mathrm{C-O(1)}}$, $d_{\mathrm{C-O(2)}}$, δ_1, δ_2, $d_{\mathrm{O\cdot\cdot N(1)}}$, $d_{\mathrm{O\cdot\cdot N(2)}}$.

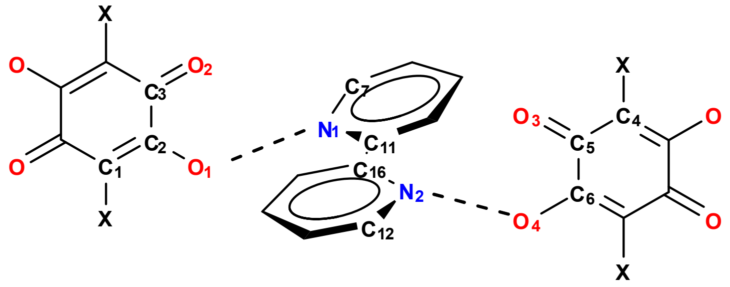

Figure S9. Definitions of chemical-bond parameters. Bond distance $d_{\mathrm{C-O(1)}}$; C_2–O_1, $d_{\mathrm{C-O(2)}}$; C_6–O_4. Bond angle, δ_1; $\angle C_7N_1C_{11}$, δ_2; $\angle C_{12}N_2C_{16}$. Hydrogen-bond distance, $d_{\mathrm{O\cdot\cdot N(1)}}$; $O_1\cdot\cdot N_1$, $d_{\mathrm{O\cdot\cdot N(2)}}$; $O_4\cdot\cdot N_2$, $d'_{\mathrm{O\cdot\cdot N(1)}}$; $O_2\cdots N_1$, $d'_{\mathrm{O\cdot\cdot N(2)}}$; $O_3\cdots N_2$.
